# A longitudinal analysis of physical exercise in shaping language learners’ emotional well-being: a comparative analysis between L1 and L2 students

**DOI:** 10.1186/s40359-024-02338-9

**Published:** 2025-01-16

**Authors:** Huma Akram, Ibrahim Naser Oteir

**Affiliations:** 1 School of International Education, North China University of Water Resources and Electric Power, Zhengzhou, China; 2 General Studies Department, Applied Science University, Manama, Bahrain

**Keywords:** Physical exercise, L1 learners, L2 learners, Emotional regulation, Process model

## Abstract

**Background:**

Students’ psychological wellness is one of the key elements that improve their well-being and shape their academic progress in the realm of language learning. Among various strategies, physical exercise emerges as an effective approach, allowing learners to manage their emotions considerably.

**Methods:**

Employing a quasi-experimental research design, this study examines the impact of a three-month physical running exercise intervention on emotional regulation behaviors among L1 (Arabic language) and L2 (English as a foreign language learning) students. Data was collected at three (pre-test, mid-test, and post-test) intervals, focusing cognitive reappraisal (CR) and expressive suppression (ES) the key constructs of emotional regulation.

**Findings:**

The results showed that the emotional regulation abilities of both groups were considerably impacted by the physical running exertion and differed significantly, with students’ CR skills significantly improving and their ES decreasing over time. However, no significant interaction effect between time and (L1 and L2) groups’ CR was observed, suggesting that physical exercise universally benefits cognitive reappraisal regardless of the language learning context. Conversely, a significant interaction effect was observed in students’ ES, with L2 students experiencing more reduction compared to their L1 counterparts, highlighting the unique emotional challenges faced by L2 learners and the effectiveness of physical activity in mitigating these challenges.

**Conclusion:**

The results highlight the importance of physical exercise in enhancing emotional regulation abilities among students, particularly in a second language learning context. Given this, regular physical activity programs should be incorporated into educational curricula to support students’ emotional well-being and academic success. It further offers insightful recommendations for teachers, students, administrators, and policymakers to optimize physical exercise integration in higher education.

## Introduction

Conforming to existing educational and behavioral research, students’ psychological wellness plays an essential role in determining how well they succeed in education and society [1, 2]. Acknowledging this recognition, the global movement has prioritized student emotional wellness in higher education with a particular consideration on emotional regulation [3, 4]. This term broadly refers to an ingenuity to deal with and address emotional situations in a healthy way [5]. At the same time, enhancing capabilities of regulating emotions is considered effective in empowering students to improve their academic performance [6], motivation [7], mental health [8], sense of happiness in life [9], and social interactions [10]. Besides, emotional competency is instrumental in addressing students’ academic issues and foster their sense of enjoyment in the process of learning [11]. Individuals who struggle to control their emotions go through a lot of unpleasant instances, which can lead to psychological disorders such as depression or anxiety [12].

Emotional competency is of utmost importance when it comes to EFL learning practices [13, 10]. Since, students from countries where English is not recognized as a second language, often face the significant challenge of limited opportunities to use English in their daily lives, which hampers their ability to practice and improve their language skills [14]. This lack of real-world practice opportunities prevents students from developing fluency and confidence in using the language in authentic contexts [15]. As a result, learning a language can occasionally be an anxious endeavor for a number of students [16], which requires to regulate properly to keep them active psychologically and progress positively. A number of studies have reported the importance of regulating emotions for language learners in managing their anxiety [12], keeping them motivated [17], and succeeding in their language acquisition [18]. Given this, language learners must know to how to control their negative emotions in order to succeed academically and develop personally.

To enhance emotional regulation skills among language learners, previous studies indicate numerous interventions, such as mindfulness practices [19], cognitive-behavioral strategies [20], social support programs [21], and creative arts therapies [22]. Meanwhile, the positive role of physical exercise on psychological and emotional well-being has been well documented in the literature [23–25]. However, there is still a gap in understanding the role of physical exercise, in regulating emotions among language learners. Moreover, emotional regulation patterns in the educational process may vary greatly across L1 and L2 learners due to differences in cognitive load [26], cultural context [27], and language acquisition experiences [15]. L2 learners often face additional stress and anxiety stemming from the challenges of learning and using a second language, which can influence their ways of regulating their emotions [28]. In contrast, L1 learners typically navigate their educational environment with greater linguistic ease, potentially leading to different emotional responses and regulatory mechanisms [29]. Understanding these variations is crucial, as it can inform tailored interventions and support strategies that address the specific emotional needs of both L1 and L2 students. Our study thus aims to examine the effects of a daily physical running exercise intervention on the emotional regulation behaviors of students majoring in Arabic (L1) and English (L2) language by seeking these objectives:

1. To explore the role of running in shaping students’ emotional regulation behaviors.
2. To compare the emotional regulation behaviors of students with respect to their majors.

## Literature review

### Emotional regulation

Dipping into the Latin word “emovere,” emotion signifies to a mode of inciting and invigorating an individual [30]. With regard to its lexical foundation, affective processes have the ability to energize an individual to move forward. Emotions, as a multifaceted construct, are made up of an extensive spectrum of interconnected psychological functions that occur simultaneously [31]. When it comes to foreign language learning, significant attention has been paid to comprehending the distinct emotions of learners. A review of the previous studies indicates that students’ language learning is tied to various emotional states, including anxiety [17], learning motivation [7], and enjoyment [32]. Dörnyei [33] further illustrates language learning an emotionally loaded process, which requires teachers to comprehend in a proper way. Learners’ emotional spectrum, as noted by Su and Guo [34], may significantly influence various aspects of their academic and emotional endeavours, such as cognitive progression [35], learning motivation [36], listening self-efficacy [37], and behavioural engagement [20].

As students go through a range of emotions during the course of their studies, they must actively handle and convey positive feelings like enthusiasm and positive attitudes [38] while also actively controlling negative emotions [10]. Echoing this perspective, research has affirmed that positive emotions such as motivation, excitement, and satisfaction are central to effective language learning/acquisition [39]. On the other hand, adverse feelings such as nervousness or stress negatively affect their psychological wellness and students learning [40]. Given this, the methods students employ to control their emotions become essential since they can greatly improve their psychological health and promote academic achievement [41]. As per Gross [5, 42], emotional regulation (ER) incorporates a variety of spontaneous or intentional steps used to deal with both positive and negative emotional situations. In other words, it is a process through which individuals give meaning to their feelings leading towards an emotional response [43]. Language learners who effectively regulate their emotions are more capable of acquiring academic success [44], social well-being [45], healthier teacher-student relationships [46], and self-directed learning skills [47]. Additionally, emotional regulation helps sustain student interest, allowing them to strengthens the effectiveness of their educational journey [34].

### Physical exercise and emotional regulation

Physical exercise is well-documented for its positive effects on mental health [48] cognitive function [23], and emotional regulation [24, 25]. Its regular adoption can lead to improvements in mood [49], reductions in anxiety and depression [50], and enhanced emotional resilience [51] in several ways. Regarding physiologically, it stimulates the release of endorphins, serotonin, and other neurotransmitters that elevate mood and reduce stress [52]. In addition, regular physical activity also helps regulate the hypothalamic-pituitary-adrenal (HPA) axis, which is involved in the body’s stress response [53, 54].

In terms of psychological benefits, physical exercises such as yoga, aerobic, or endurance help improve mood [55] release stress [50], enhance self-esteem [56], and develop a sense of accomplishment [57], which contribute to better emotional regulation [58]. Likewise, Giles et al. [59] highlight the significance of physical exercise by denoting its positive effects on individuals, where they reinterpret stressful situations in a more positive manner. Engaging 61 participants in an experimental study, Morello et al. [60] found a positive association of aerobic exercises with coping mechanisms and emotional regulation behaviors. Linking physical exercise with stress management, Tong et al. [61] identified a significant role of yoga and mindfulness in coping with the stress. In another study, Lu [62] surveyed 386 undergraduate students from a Chinese college and found a negative correlation between physical activity with resilience and negative emotions. Taking together, the above-mentioned studies divulge that physical exercise plays a crucial role in students’ emotional regulation in learning environments. Despite such attempts, the examination of physical exercise in relation to language students’ emotional regulation is still in its infancy. Moreover, the research on comparison between L1 and L2 learners is yet to be investigated. To fill this lacuna, this research aims to observe the influence of a daily running exercise on the emotional regulation behaviors of students majoring in Arabic (L1) and English (L2).

### Theoretical underpinning

Building upon the objectives of the study, this study adopts Gross’s [42] process-oriented model of emotion regulation (ER) to explain the patterns of ER behaviours among language learners. This model delineates two distinct temporal approaches that offer a structured understanding of the dynamics of the emotion management process.

#### Cognitive reappraisal

Cognitive reappraisal (CR) encompasses the purposeful reinterpretation of a situation that elicits emotions, leading to a transformation of its significance and a modification of its emotional impact [63, 42]. An examination of the chronological progression of emotions indicates that cognitive reappraisal exerts its primary influence at specific phases within the emotion-generation process [64]. To be more specific, CR occurs prior to the complete activation of emotional responses, potentially reshaping the entire course of the emotional response before it fully materializes. As a result, it is often termed “antecedent-focused regulation.” When it comes to language learning, students who are capable of reinterpreting challenges as opportunities for personal growth may exhibit greater adaptability when facing learning setbacks [65]. This adaptability is likely to reduce the anxiety of learning new language and enhance confidence among them. In addition, the individuals who exercise every day are more likely to use CR [60], as exercise improves mood and enhances students’ ability to reframe challenging language learning situations. Thus, following hypotheses can be put forth in light of the context of our study:

1. Running intervention significantly enhances students’ cognitive reappraisal (CR) skills over time.
2. The effect of running intervention on CR significantly differs between L1 and L2 students.

#### Expressive suppression

The second strategy expressive suppression (ES) represents a response-focused strategy, intervening once emotions are already in progress and after behavioural responses have fully materialized [64]. Consequently, it may necessitate recurring efforts to manage emotional responses as they persistently emerge, placing demands on an individual’s cognitive resources [66]. Besides, response-focused regulation comes into play after emotions have fully developed. In this approach, individuals attempt to regulate their emotions by modifying any of the components that constitute the emotional experience [67]. In particular, it may involve altering facial expressions, adjusting vocal tone, suppressing thoughts, regulating physiological arousal, and even changing subjective feelings [64]. Broadening this theory, Zheng and Zhou [32] specify the adverse effects of ES on EFL learners. They noted that students who employ ES, they may inadvertently increase their stress levels and diminish their problem-solving capabilities, particularly when faced with the challenges in language learning. In relation to physical exercise, while ES is generally less adaptive than CR, physical exercise can still play a role in moderating its effects [68]. Extending this concept to university students, Lu [62] pointed out that exercise can lower the frequency and intensity of negative emotions that learners might feel compelled to suppress by reducing overall stress. In a similar vein, Al-Wardat, et al. [69] also observed that the increased emotional resilience gained from regular exercise can help learners find more adaptive ways to express and manage their emotions, reducing the need for suppression. Given this backdrop and the context of our study, the following hypotheses can be proposed.

3. Running intervention significantly reduces students’ expressive suppression (ES) skills over time.
4. The effect of running intervention on ES significantly differs between L1 and L2 students.

## Methodology

Employing a quasi-experimental research design, we implemented a daily running exercise intervention at a Bahraini university to investigate its role in students’ emotional regulation skills. Following a non-equivalent group protocol, 300 students from second-year cohorts were selected through convenience sampling. Based on their majors, students were classified into two groups, i.e., L1 and L2, comprising 150 students each. The L2 group involved students majoring in English as a foreign language with high proficiency in being exposed to English academically. These students had studied intensive English for at least two years at the university level. On the other hand, the L1 group was majoring in Arabic language studies with little to no exposure to English save for basic courses that the university mandated. The participants were aged between 18 and 22 years (mean age = 20.2 years), including 98 females and 202 males. To gain a homogeneous population, we made sure that participants had no experience of daily running to check the actual effect of the intervention. In order to confirm this, all the participants were interrogated via a self-reported survey before starting the study. In addition, the demographic characteristics of both groups were also compared using chi-square and t-tests’ analyses, and the results are presented in the results’ section.

The intervention lasted for three months, during which all participants engaged in a 30-minute running session every day. Attendance was monitored to ensure compliance, and participants were encouraged to maintain a consistent exercise routine. This approach allowed us to compare the effects of the exercise intervention between the two groups effectively. All participants participated in the study voluntarily and were informed of their right to withdraw at any time. The online survey included a cover letter explaining the study’s purpose and the participants’ rights. In addition, scholarly research and the ethics board of a Bahraini University also gave their permission to conduct the study. Thereby, all the procedures were compliant with the applicable regulations in accordance with Helsinki’s guidelines pertaining to human subjects.

### Measure

Students perceived capabilities of regulating their emotions were longitudinally assessed via questionnaire at three different stages: before, mid, and after the intervention to understand how their emotional regulation skills change across different intervals. The structured survey was adopted by Preece et al.’s [70] scale based on the process model framework. It has been authenticated by many researchers across diverse contexts, such as Smith et al., [71] in the United States and Mazidi et al. [72] in Iran, demonstrating the scale’s robustness. It was modified to meet the study’s context and consists of 10 items, CR with six items, and ES with four items. All questions were rated on a 5-point Likert scale, from low level of discordance to high level of accordance. To ensure content quality and consistency, professional linguists initially reviewed the questionnaire to verify participants’ comprehension and response accuracy [73]. It was further gone through a meticulous translation and back-translation process for verifying its appropriateness in the Bahraini context. Subsequently, the survey was distributed to study participants via the Google Docs platform. Participants received reminders to complete the surveys at each assessment point (pre-test, mid-test, post-test). We then assessed the scale’s internal consistency to guarantee reliability in the local setting, which is explained in the following section.

## Results and data analysis

### Baseline comparison of groups

To ensure the baseline comparability, both of the groups were first compared using chi-square and t-tests, with respect to their demographic features and emotional regulation levels prior to the intervention (see Table 1). The analysis revealed no significant difference across age (*t* = -0.79, *p* = .22), gender (*χ*² = 0.41, *p* = .42), and emotional regulation constructs, i.e., CR (*t* = -1.25, *p* = .13) and ES (*t* = -0.92, *p* = .26) suggesting that both groups were demographically equivalent, and ready to be participated in the study.

**Table 1:** Groups’ comparison matrix

Variables	L1	L2	(t/χ2)	*p*-value
	Mean (SD)	Mean (SD)		
Gender	65%	64%	0.41	0.42
Age	21.2 (1.3)	21.3 (1.4)	-0.79	0.22
CR	2.63 (0.68)	2.71 (0.74)	-1.25	0.13
ES	3.51 (0.77)	3.58 (0.92)	-0.92	0.26

### Model consistency

The adequacy of the model was then assessed across both samples prior to data analysis (see Table 2). The goodness-of-fit indices suggested that the model had an acceptable fit to the data across both samples [74], which confirmed that the factor structure of the survey instrument was robust and reliable.

**Table 2:** Model fit indices

Fitting indices	Recommended values	Model values for L1 students’ sample	Model values for L2 students’ sample
*χ2/ df*	< 3	2.51	2.62
RMSR	< 0.08	0.071	0.75
RMSEA	< 0.08	0.067	0.072
CFI	> 0.90	0.92	0.94
GFI	> 0.90	0.93	0.94
AGFI	> 0.8	0.83	0.86
TLI	> 0.90	0.91	0.93

The validity and reliability of each parameter across both samples were checked to ensure that the data corresponded to the study model (see Table 3). Using Cronbach’s alpha, we tested the dependability of each parameter, which yielded values greater than 80%, indicating sufficient convergence to continue data analysis [75]. The validity was assessed using convergent validity via composite reliability (CR) and average variance extracted (AVE), which also yielded robust values i.e., CR > 0.70 and AVE > 0.50, respectively [76]. The factor loadings further elaborated the construct validity and yielded strong loadings of all the items [77], and discriminant validity of all the constructs [74].

**Table 3:** Reliability and validity matrix

Variables	Items	Loadings	Alpha Value		CR	AVE	DV
**L1 Students’ Sample**							
Cognitive reappraisal (CR)	CR1	0.79	0.83		0.87	0.70	0.83
	CR2	0.78					
	CR3	0.82					
	CR4	0.81					
	CR5	0.80					
	CR6	0.81					
Expressive Suppression (ES)	ES1	0.80	0.82		0.88	0.73	0.85
	ES2	0.81					
	ES3	0.82					
	ES4	0.81					
**L2 Students’ Sample**							
Cognitive reappraisal (CR)	CR1	0.78	0.81	0.87		0.72	0.84
	CR2	0.79					
	CR3	0.80					
	CR4	0.80					
	CR5	0.81					
	CR6	0.80					
Expressive Suppression (ES)	ES1	0.79	0.82	0.88		0.70	0.85
	ES2	0.80					
	ES3	0.81					
	ES4	0.81					

### Data analysis

The survey data was then analyzed using descriptive and inferential statistics. Descriptive statistics summarized students’ perceived ER capabilities across both dimensions at three time-intervals, i.e., pre, mid, and post-test stages. The analysis revealed notable changes in mean scores of students’ perceived ER capabilities over the course of the intervention (see Table 4). At the pre-test stage, both CR and ES levels were relatively low among both L1 and L2 students, with L2 students exhibiting slightly higher initial levels. As the intervention progressed, mid-test results indicated a moderate increase in CR across both groups, with L2 students consistently displaying higher mean scores (2.81) than their L1 students (2.69). This upward trend in CR continued through to the post-test stage, with L2 students showing the most significant increase, suggesting that these students were better able to reinterpret and manage stressful situations effectively.

Conversely, ES levels demonstrated a different pattern. While both L1 and L2 students started with relatively high levels of expressive suppression, i.e., 3.51 and 3.58 respectively, mid-test results showed a slight decrease in ES among both groups with mean scores 3.21 and 3.23 L2 respectively. By the post-test stage, this decrease became more pronounced for L2 students (2.83) than the L1 students (2.78), indicating a reduction in the tendency to suppress emotions. This decrease suggests that L2 students were becoming more comfortable with expressing their emotions rather than suppressing them, likely due to the positive impact of the intervention.

**Table 4:** The descriptive statistics across three time-intervals

Time interval	Groups	CR		ES	
		M	SD	M	SD
Pre-Test	L1	2.63	0.68	3.51	0.77
	L2	2.71	0.74	3.58	0.92
Mid-Test	L1	2.69	0.79	3.21	0.85
	L2	2.81	0.76	3.23	0.82
Post-Test	L1	2.98	0.88	2.78	1.03
	L2	3.11	0.84	2.83	0.94

**Fig. 1:**
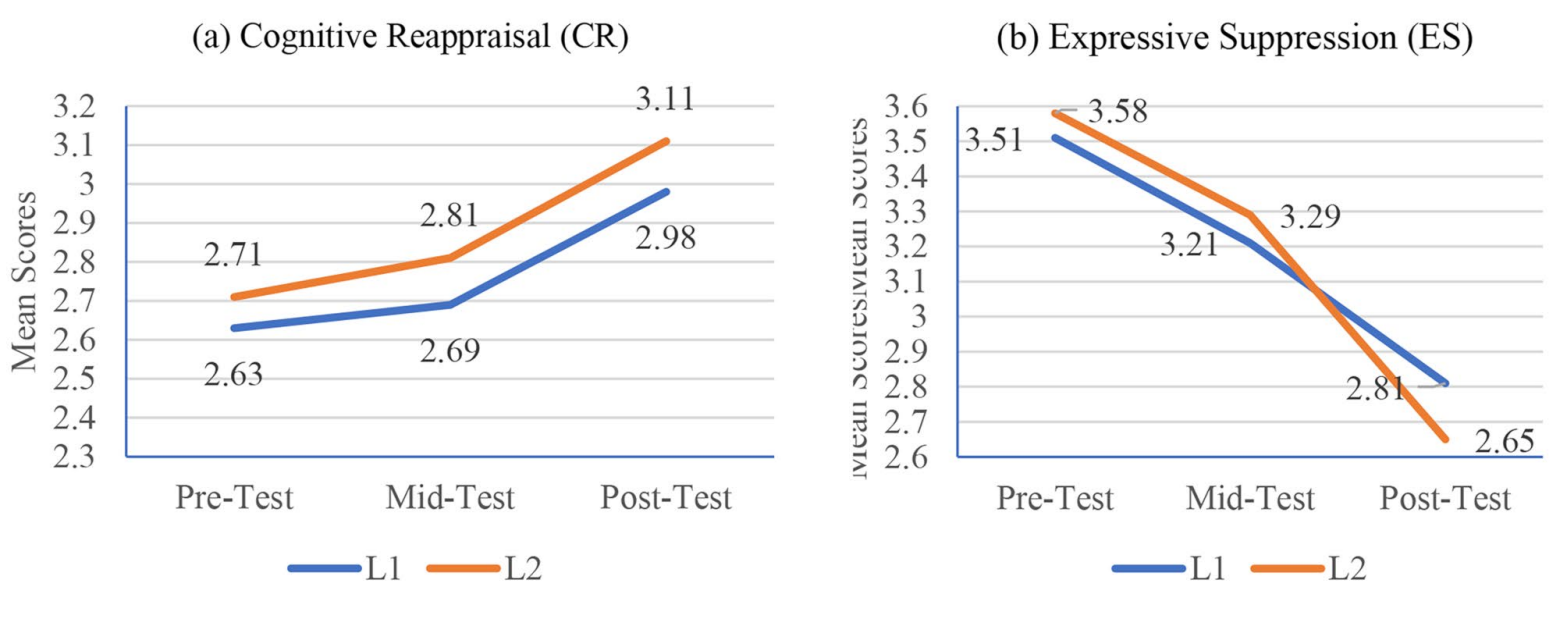
Mean scores across different time points. (**A**) Mean scores for Cognitive Reappraisal for L1 and L2 students across time. (**B**) Mean scores for Expressive Suppression for L1 and L2 students across time

To compare the differences between both groups and effect of time across ER’s dimensions, repeated measures ANOVA was conducted (see Table 5). The analysis revealed a significant interaction effect between CR and time, *F*(2, 298) = 13.23, *p* < .001, indicating that cognitive reappraisal levels significantly increased from pre-test to post-test, thereby supporting hypothesis 1.. Both groups also demonstrated a significant difference concerning CR, *F*(1, 144) = 8.45, *p* = .03, with L2 learners showing higher scores than L1 learners, thus supporting hypothesis 2 (see Fig. 1). However, no significant interaction effect was found between time and group, *F*(2, 298) = 3.62, *p* = .12, indicating that L1 and L2 students’ CR did not differ substantially over time.

**Table 5:** Repeated measures ANOVA results

Key Constructs	Effect	F	df1	df2	*p*	Partial η²
Cognitive Reappraisal (CR)	Time	13.23	2	298	0.000	0.08
	Group (L1 vs. L2)	8.45	1	149	0.03	0.05
	Time × Group	3.62	2	298	0.12	0.03
Expressive Suppression (ES)	Time	9.32	2	298	0.000	0.04
	Group (L1 vs. L2)	4.15	1	149	0.04	0.06
	Time × Group	5.12	2	298	0.01	0.03

For ES, the repeated measures ANOVA also showed a significant main effect of time, *F*(2, 298) = 9.32, *p* < .001, indicating that expressive suppression levels significantly reduced over the intervention period, thus validating hypothesis 3. Both groups also demonstrated a significant difference concerning ES, *F*(1, 144) = 4.15, *p* = .04, with L2 learners showing a greater reduction than L1 learners, aligning with hypothesis 4. Similarly, we also observed a significant interaction effect between time and group, *F*(2, 298) = 5.12, *p* = .01, indicating that L1 and L2 students’ ES differed substantially over time.

## Discussion

Language learners encounter a variety of emotions throughout their academic journey, ranging from positive to negative experiences. If they learn to manage their emotions, it can considerably improve their well-being and academic progress. Various strategies have been proposed to help students better manage their emotions. Using the Process Model as a framework, this study delves into the ways in which physical running exercises affect language learners’ emotional regulation behaviors, via cognitive reappraisal and expressive suppression strategies.

Using a quasi-experimental research design, the findings indicate that the physical running exercise significantly affects both L1 and L2 students’ emotional regulation skills. In terms of the first strategy, students’ CR skills were found significantly increased by regular physical running exercise over time. This insight corresponds with the observation of Giles et al. [78], which specify that habitual exercise positively affects individuals’ capabilities in cognitive control of emotions. In agreement with this finding, Yang et al. [79] emphasize the effective role of physical activity in improving university students’ stress resilience and cognitive reappraisal. Notwithstanding, according to Perchtold-Stefan et al. [80], physical activity and individuals’ reappraisal ideas did not appear to be related, and showed association with the positive interpretations. Thus, the proposal of various forms of workouts or exercises in the syllabus might be perceived as a useful approach to enhancing emotional regulation in language learners. It should also be advocated by teachers that students take an interest in participation. They also need to be aware of the emotional factors associated with language learning and give them support by helping them manage their feelings. In the development of CR strategies, teachers need to lead students to regulate their emotions and then assist students in using different types of cognitive strategies to regulate their emotions.

In terms of difference, both groups differed significantly across CR strategy. This result coincides with results of Bahmani et al. [58], who found that while physical activity tends to be positively associated with more adaptive emotional regulation strategies, the effect may vary across different groups or levels. This effect suggests of considering group-specific differences when promoting adaptive emotional regulation strategies. However, the absence of a significant interaction effect between time and group indicates that the rate of change in CR over time did not differ between L1 and L2 groups. In this regard, educational institutions should universally promote physical activity as part of their academic programs and encourage all students to engage in regular exercise to enhance their emotional regulation capabilities.

Regarding the second strategy of regulating emotions, students’ ES was found significantly decreased by regular running exercise over time. This insight reflects Gross and Cassidy’s [67] insights, who considered ES a less adaptive emotional regulation strategy compared to cognitive reappraisal. This finding echoes the finding of Wu et al. [81], who identified a significant negative correlation of physical activity with ES strategy among college students. However, Al-Wardat et al. [69] found no significant association between physical activity and suppression practices among individuals, but physical activity significantly decreased the level of both depression and anxiety. Considering this, teachers should leverage the healthy landscape of the physical activity to facilitate students learn better stress management and overall emotional well-being. They should further encourage students to express their emotions openly instead of suppressing them. It is only possible when teachers make a supportive and friendly bond with their students and help recognize and address their emotional pressures and problems. Akram and Li [82] also identify a key role of positive teacher-student relationships in students’ emotional well-being. Considering this, teachers should show empathy, listen actively, and provide guidance to students who may be struggling emotionally. Moreover, concerned authorities should develop department-wide initiatives to promote physical exercise among students as a part of their curricular or co-curricular activities. Likewise, providing professional development support for teachers may enable them to implement classroom practices and after-school activities that are beneficial for students’ emotional health.

Dealing with differences among students, both groups showed a significant difference with ES strategy. Al-Wardat et al. [69] also advocated regular physical exercise as a predictor of decrease in use of suppression and increase in expression of emotions. Our data support this idea by showing that the reduction in suppression after the running intervention was larger among L2 learners. Possibly because L2 students experience high levels of pressure to perform academically in a second language [83], which increases their susceptibility to suppression as a coping strategy [84]. In light of that, educational institutions should possibly implement frequent physical fitness programs throughout the curriculum. Likewise, maladaptive emotional regulation strategies, such as suppression, can be reduced by providing them with opportunities for physical activity to attain better mental health outcomes. However, Chen et al.’s [85] observation imply that ES can have positive emotional regulation effects among Chinese individuals. Thus, future studies should examine the ways ES shapes language learners’ emotional well-being in navigating dual cultural expectations. Examining these concerns would help in comprehending how cultural contexts influence emotional regulation techniques and their possible implications for the psychological and social adjustment of bilingual students.

## Conclusions

The study’s findings underscore the significant role of physical exercise in enhancing emotional regulation among language learners. It also provides a theoretical basis for updating emotion regulation models in light of physical exercise by providing a unique perspective that cultural and linguistic factors are important in defining learners’ emotional regulation capabilities. Process model on the other hand underrepresents how learners’ linguistic and cultural backgrounds influence the adaptive or maladaptive nature of emotional regulation strategies. Besides, the intervention led to a marked decrease in expressive suppression (ES) and increase in cognitive reappraisal (CR) among L2 students, highlighting the specific benefits of physical activity for those facing the unique stressors of learning in a second language. These changes suggest that physical exercise can effectively address the heightened emotional challenges experienced by L2 learners, promoting healthier emotional regulation strategies. In contrast, the lack of a significant interaction difference in cognitive reappraisal (CR) between L1 and L2 students indicates that the benefits of physical exercise on CR are uniformly distributed across different language learning contexts. This uniform improvement suggests that physical activity is a broadly applicable intervention for enhancing adaptive emotional regulation strategies, regardless of the specific language learning environment. Given these findings, there are some important implications for educational practices. Integrating regular physical exercise into the curriculum can provide substantial benefits for students’ emotional regulation, particularly for L2 learners who face additional language-related stressors. Moreover, fostering positive teacher-student relationships is crucial for recognizing and addressing students’ emotional problems, ensuring they receive the necessary support to develop effective emotional regulation strategies.

## Data Availability

The datasets generated and analyzed during the current study are available from the corresponding author on reasonable request.
